# Supplementary figures and images for: Interaction of SARS-CoV-2 Nucleocapsid Protein and Human RNA Helicases DDX1 and DDX3X Modulates Their Activities on Double-Stranded RNA
Source: Int J Mol Sci. 2023 Mar 17;24(6):5784. doi: 10.3390/ijms24065784 (PMC10058294; doi:10.3390/ijms24065784)

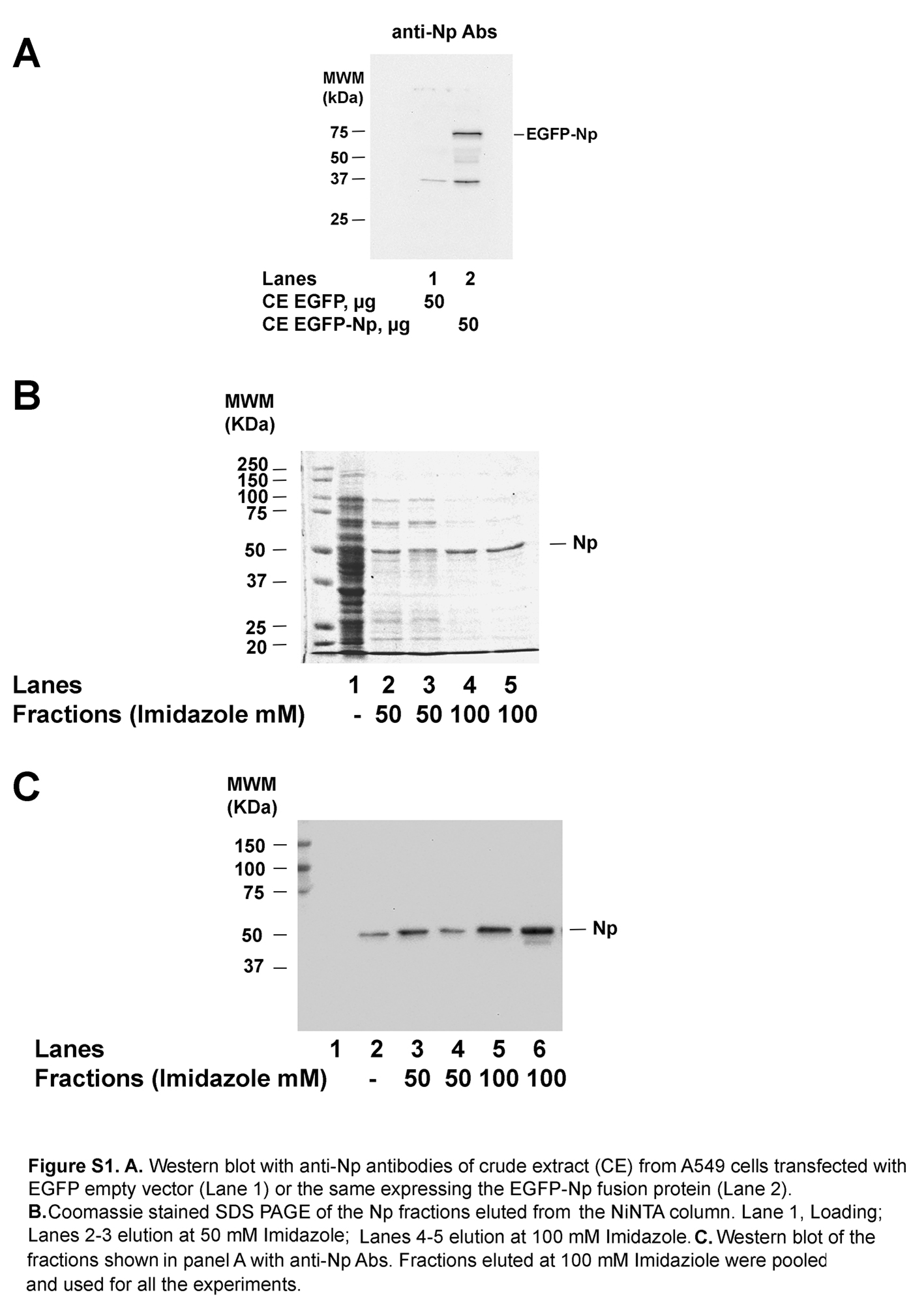

Supplement: Supplementary file 1 [file ijms-24-05784-s001.zip › Supp_Figure S1.png]

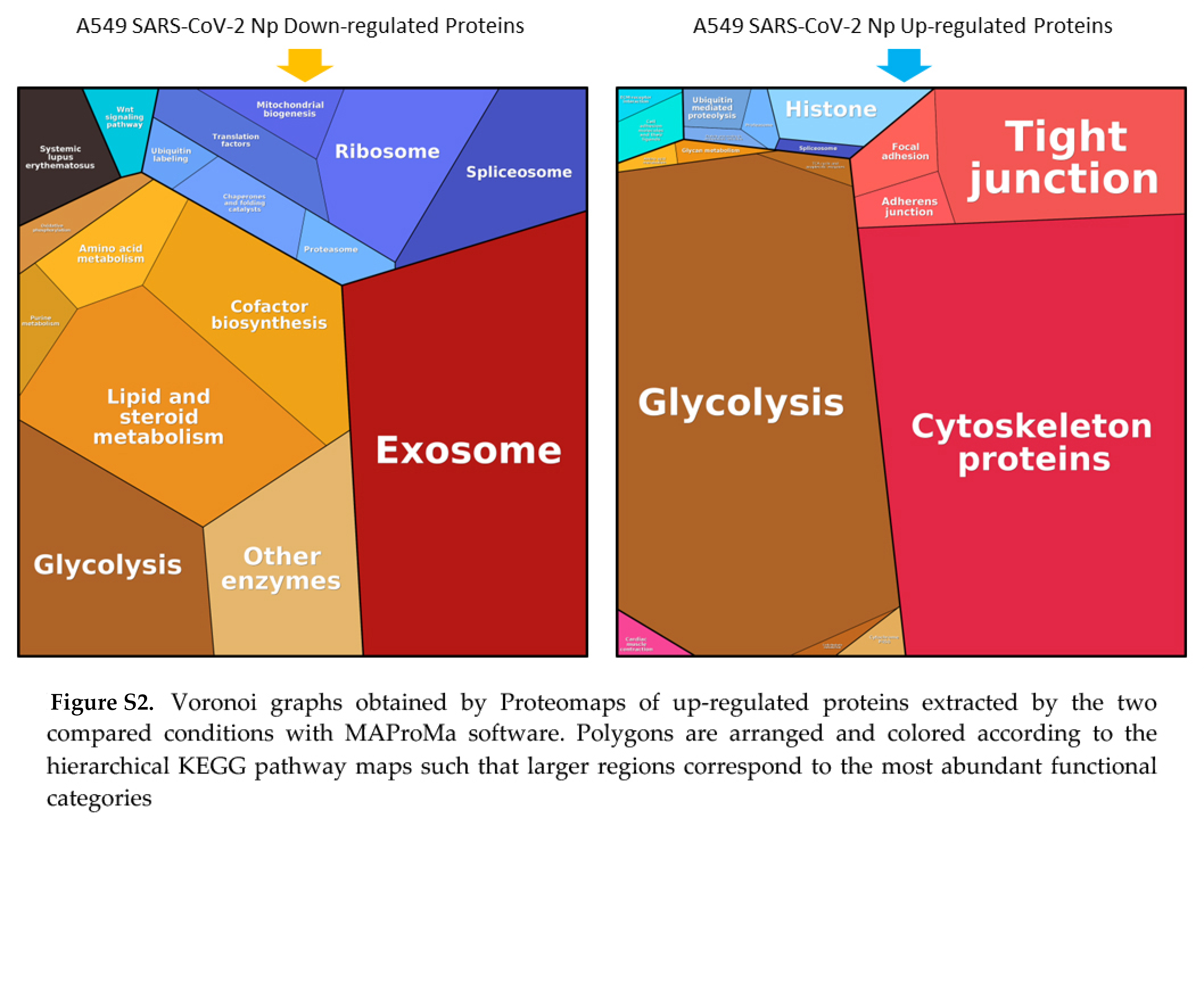

Supplement: Supplementary file 1 [file ijms-24-05784-s001.zip › Supp_Figure S2.png]

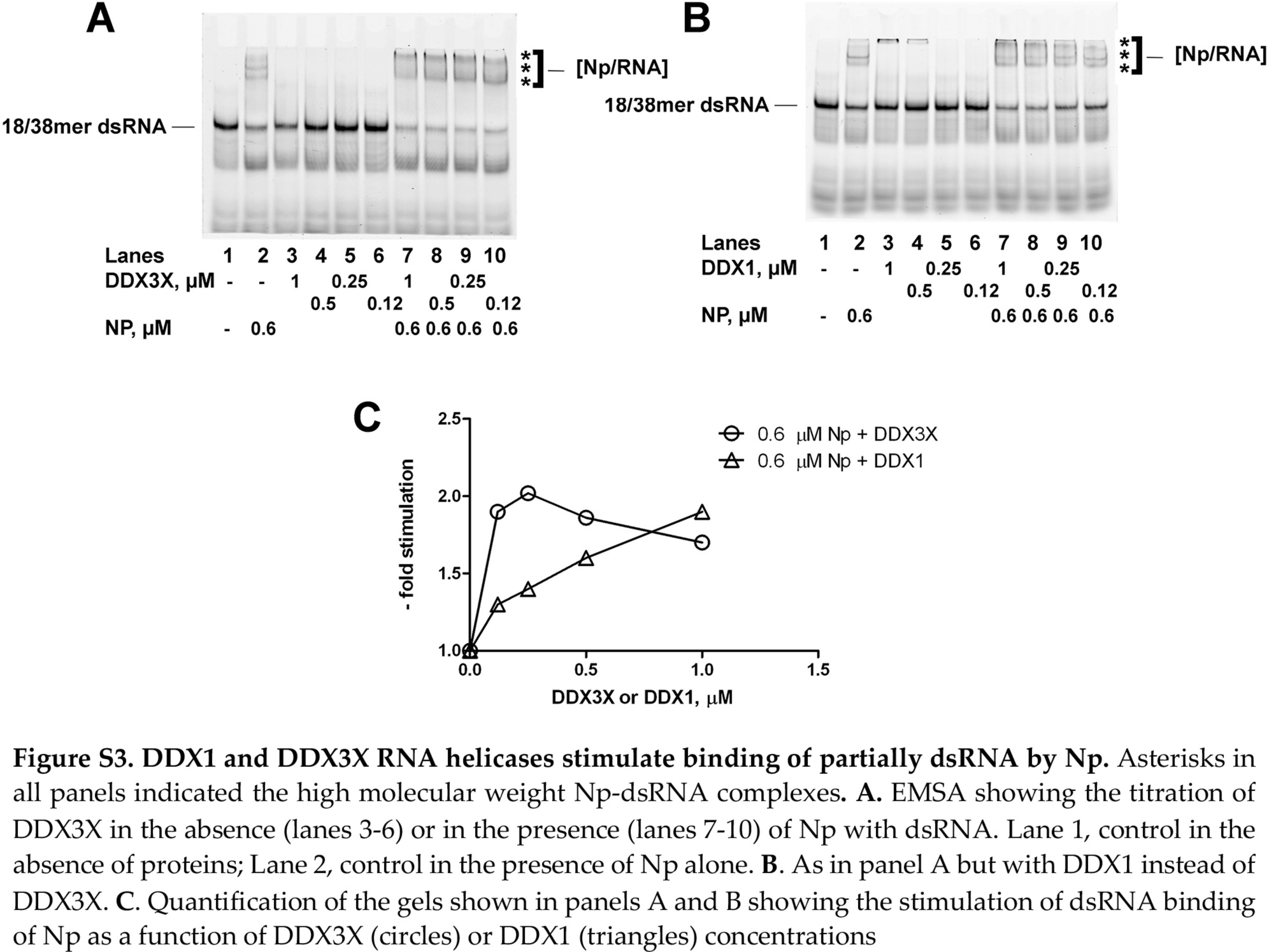

Supplement: Supplementary file 1 [file ijms-24-05784-s001.zip › Supp_Figure S3.png]

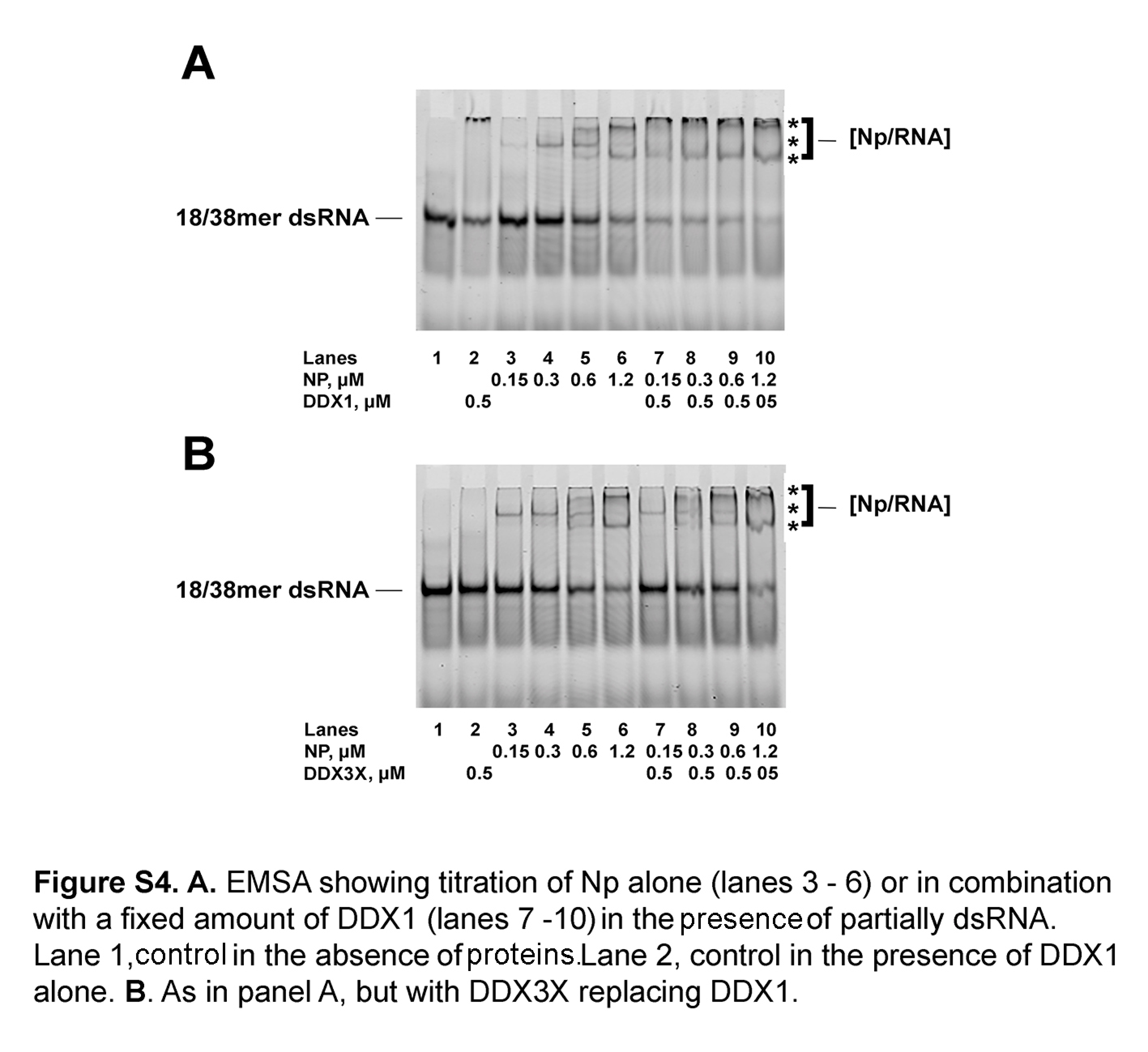

Supplement: Supplementary file 1 [file ijms-24-05784-s001.zip › Supp_Figure S4.png]
